# Strategy Adjustments of the United States and the European Union vis-à-vis China: Democratic Global Power Identities and Fluid Polygonal Relations

**DOI:** 10.1007/s11366-022-09794-3

**Published:** 2022-03-18

**Authors:** Nele Noesselt

**Affiliations:** Institute of Political Science & IN-EAST, University of Duisburg-Essen, Forsthausweg 2 (room LE709b), D-47057 Duisburg, Germany

**Keywords:** China, EU, Hong Kong SAR, Indo-Pacific, Strategic triangle, Taiwan, US

## Abstract

How are the European Union and its individual member states positioning themselves within the intensifying trade war and power struggles between Washington and Beijing? Does the fact that both the United States and the EU have recently updated their strategic approach to China and continuously underline their democratic regime patterns and support for like-minded systems—contrasted with Beijing as a perceived promoter of an illiberal world order—imply a return of “Cold War” system antagonism? Could this also result in a reemergence of strategic triangles? Shedding light on Washington’s expression of active support for Taiwan (and Hong Kong) and EUrope’s related position statements, this article concludes that these emerging constellations would best be described as strategic polygonal relationships. While the EU Commission seeks to formulate a common foreign and security strategy, the various EU member states are defining their distinct positions within the new global power matrix emerging between Washington and Beijing.

## Introduction

This article examines the positioning of EUrope^1^ in the intensifying power struggle between Washington and Beijing, reflecting on the related narrative of a revived and evolving democracy-autocracy antagonism in world politics. Is there a new (pro-)democratic transatlantic coalition emerging, one countering the rise of autocratic regimes—namely China and Russia?

To assess the changing relational patterns between EUrope, the US, and the People’s Republic of China (PRC), this article analyzes developments and position changes in the years from 2016 (US presidential election, incoming Donald Trump administration) to 2021 (inauguration of the Biden administration; reconfirmation of transatlantic security cooperation between the US and Europe). The article looks at two concrete cases: the US’s and EUrope’s stance vis-à-vis Taiwan and Hong Kong as well as their positioning in the Indo-Pacific. The dataset compiled to assess these positions includes government speeches, national (defense) strategy papers, and select background analyses by policy advisers and political scientists. Furthermore, given that political decision-making is based on the anticipated and perceived positions and strategic moves of the other players involved, the article also incorporates surveys mapping EUrope’s views on China as well as on the US. Observing that individual EU member states sometimes deviate from Brussels’ official positions, this article concludes by classifying the interactions between the US, China, and EUrope not as triangular ones but as dynamically fluctuating polygones.

## Strategic Triangles and Role Identities

How do states position themselves within the power competition between two great powers? Are actors with comparatively minor military capacities resorting to balancing or bandwagoning strategies? Assessing the role of lesser powers caught in the power struggles of two superpowers, Wu Yu-Shan postulates that secondary (or tertiary) powers would generally resort to a strategy of hedging [107], which is conceived as states’ strategic response to a rising power before the backdrop of uncertainty. It is often conceptualized as risk management based on a mixture of cooperative and confrontational behavior, merging elements of balancing and bandwagoning [15]. While cooperation with a rising power might offer economic benefits, the latter’s increase in power might also be perceived as posing a challenge to national security interests, implying that minor powers might strengthen their alliances with third parties and engage in balancing actions [107]. Wu’s analysis is based on the concept of the strategic triangle [106, 107], once applied, back in the 1970s, by Lowell Dittmer to the analysis of interactions between the PRC, the US, and the Soviet Union. Dittmer defined these interactions as “a sort of transactional game among three players” [20:485], underscoring the fluidity of triangular relations over time. According to the game-theoretical imagination of PRC-related strategic triangles, four basic types can be differentiated [20:489; 107:201]:

1. ménage à trois = a strategic triangle composed of three amity-based, symmetrical bilateral relationships;
2. marriage = one friendship-based bilateral relationship, with both partners having negative, enmity-style relations with the third party involved (as exemplified by Sino-Soviet amity and both players’ enmity towards the US between 1949 to 1960);
3. romantic triangle = friendly relations between one pivot and two wings, which are caught in an enmity-based relationship vis-à-vis one another (e.g., the US pivot position vis-à-vis the PRC and the Soviet Union in the aftermath of the Sino-Soviet split);

and, though a rather exceptional case,

4) unit veto = composed of three negative bilateral relations between three players which perceive of one another as enemy, rival, or foe.

Apart from great strategic triangles between predominant global players, world politics consist of multiple, partly overlapping, dynamically evolving, triangular relationships, as Dittmer’s study on Taiwan’s “minitriangle” illustrates. With the collapse of Soviet socialism and the transformation of Eastern European communism, the great China-US-SU triangle ceased to exist—implying a position adjustment of the US towards Beijing. The Tian’anmen incident of 1989 and the PRC’s uninterrupted commitment to socialism, however, fueled negative perceptions of communist China. The then-elected George W. Bush administration intensified its symbolic support for Taiwan and downgraded the PRC from “strategic partner” to “strategic competitor.” While, according to Dittmer, after 1989/1991, the US became the pivot in the emerging equilateral China-US-Taiwan minitriangle, the ongoing tensions and enmities between the two Chinese wings of the triangle implied a permanent risk and factor of incalculability [21]. Back in 2002, Thomas Christensen, assessing the Taiwan issue, argued [14] that the US was walking a dangerous tightrope in seeking to find a balance between deterrence and reassurance. Similarly, Pan Zhongqi’s study of the US’s strategic ambiguity toward Taiwan [67] as well as the summary by Tucker and Glaser of the debates in the US about the costs and risks resulting from the Taiwan Relations Act [98] document that two decades ago—at the turn of the new century—the US’s strategic reflections included the idea of reducing strategic-symbolic support for Taiwan as this was seen as a major obstacle to the deepening of trade between the US and the PRC. However, the expected democratic transformation of the PRC via active engagement did not occur. And, following Beijing’s accession to the World Trade Organization and its rise to the world’s second-largest economy, the PRC’s powerful state-controlled capitalism is now perceived as posing a direct challenge to US global trade interests and the US-centered international order. These perception (and strategy) changes have, as this article will show, triggered a reconfirmation of the autocracy-versus-democracy paradigm in US foreign policy and resulted in a novel assessement of Taiwan within Washington’s Indo-Pacific strategy and the related strategic triangles.

Guided by the concept of the strategic triangle, recent scholarly research on the EU’s—or leading EU member states’—positioning between Washington and Beijing has ascribed EUrope a strategic pivot position [95]. In this vein, Sebastian Biba’s study on Germany’s positioning vis-à-vis the US and the PRC in the Trump years underlines that the widely-predicted “ganging up on Trump”—e.g., via the formation of a strategic Sino-German alliance seeking to counter the US “America First” principle—did not materialize [6]. Looking at transatlantic relations under the Biden administration, Biba even assumes a transformation of the US-Germany-China (PRC) triangle towards a more or less stable transatlantic “marriage” [7].

This article, adding Taiwan to the equation, proposes a further modification of the strategic triangle by undertaking a role-theory-based reading [43]. To assess the positioning of EUrope within the aggravating competition for global power and status between Washington and Beijing, it is necessary to identify the images, roles, and strategic positions EUrope associates with these two great powers and to shed light on the related minitriangle with Taiwan. Apart from the roles and images ascribed to others, the positioning within the strategic triangle also contains the layer of actors’ self-proclaimed role-identity. According to Anthony Giddens’s concept of “ontological security,” actors are expected to strive for a “sense of continuity and order in events” [41:243].^2^ Their social (role-)identity is based on the “capacity to keep a particular narrative going” [41:54] and the ability to reiterate symbolic “routines” [41:44, 167]. The images EUrope ascribes to the US and the PRC, as this article argues, are formed not only by concrete interactions or the assumed power capacities and strategic positions of the other(s) but mirror states’ self-proclaimed roles and (regime-type-related) identity narratives. The observed global re-strengthening of autocratic regime patterns in combination with the EU’s internal frictions and regional security challenges in Europe have resulted in a re-evaluation of role-identity claims as well as of the role-ascriptions towards (significant) others.

The following sections of this article will take a closer look at EUrope’s China strategy before the backdrop of the dynamically evolving transatlantic partnership between Washington and Brussels. The article will hence not examine the concrete actions or the diplomatic statements voiced by the PRC but evaluate EUrope’s responses and reactions to Washington’s China (and Taiwan) strategy and the classification of Beijing as a potential challenger to “Western” democracy.

## Mapping the US Position

The US’s rhetorical opposition to the PRC is connected to a narrative of an inevitable conflict between the liberal world order and a rising “illiberal” counterpart—allegorically illustrated by the Thucydides trap [2, 11] in which the US and China might presumably be caught. The 2017 US National Security Strategy listed the PRC as a “revisionist power” [87]; the 2018 “Summary of the US Defense Strategy” labeled China a “strategic competitor” [99]. On October 4, 2018, Vice President Mike Pence highlighted the failure of the US “regime change via engagement” approach and stressed that the US and China had entered into a “new era of ‘great power competition’” [47]. Moreover, he underlined that the PRC’s success in convincing Taiwan’s few remaining supporters to switch diplomatic recognition to Beijing instead would “threaten the stability of the Taiwan Strait” [47]. While Pence confirmed official US adherence to the One-China policy and the Taiwan Relations Act, he also stated that “America will always believe that Taiwan’s embrace of democracy shows a better path for all the Chinese people” [47].

The PRC is hence ascribed the role of an antagonist “communist” player challenging the liberal democratic order, as stressed again in a speech delivered by US Secretary of State Mike Pompeo on July 23, 2020, entitled “Communist China and the Free World’s Future.” This speech condemned Beijing’s acts of “repression in Hong Kong and Xinjiang” and framed China as a “Frankenstein” monster, juxtaposing the US “way of life” and the “free world” with China’s “new tyranny” [68]. Pompeo’s Frankenstein metaphor symbolically acknowledged the failure of the US neoliberal engagement strategy [109]—and paved the way for a shift from engagement to active competition and deterrence. Bringing regime antagonism and system rivalries back in, Pompeo also postulated that China’s rise would put Asia’s democracies at risk and that it would hence be the US’s duty to intervene in defense of its democratic allies in Asia: “We marginalized our friends in Taiwan, which later blossomed into a vigorous democracy […]. Look at the Hong Kongers clamoring to emigrate abroad as the CCP [Chinese Communist Party] tightens its grip on that proud city. They wave American flags” [68].

As a preemptive move to oppose any potential efforts by Beijing to oversee a reunification with Taiwan—a strategic goal associated with certain messages sent by Xi Jinping, and, finally, directly voiced in his speech delivered on the occasion of the CCP’s 100th anniversary [108]—in March 2018 Trump signed the Taiwan Travel Act into law [85], legalizing the direct travel of high-level officials between the US and Taiwan. Subsequently, in 2019, Tsai Ing-wen traveled to New York and Denver to meet with United Nations delegations from those states that grant(ed) Taiwan diplomatic recognition [42]. In October 2019, the US Senate passed the Taiwan Allies International Protection and Enhancement Initiative (TAIPEI Act), which was finally approved by the House of Representatives on March 4, 2020. With this initiative, the US confirmed its backing of Taiwan’s quest to secure (new) international allies [84]. The drafting of the TAIPEI Act, associated with the pro-Taiwan camp around US Senator Cory Gardner, happened after several states had switched diplomatic recognition to Beijing [16]. This shift added to the narrative of a silent expansion of China’s (“authoritarian”) sphere of influence and the related erosion of US power and global status. In light of the spreading SARS-CoV-2 virus, the US, once again, expressed its support for Taiwan’s efforts to become a full member of the World Health Organization (opposed by Beijing, as membership requires statehood). Several high-ranking US politicians, including Health Secretary Alex Azar and Under Secretary of State for Economic Growth, Energy, and the Environment Keith Krach, paid official visits to Taiwan (August/September 2020) [104].

The reconfirmation of cooperation with Asian democracies also included symbolic backing for Hong Kong. Reacting to the passing of the National Security Law by the National People’s Congress (June 30, 2020), on July 14, 2020, Trump signed an order that formally ended the preferential, special status of Hong Kong. Trump’s Executive Order 13936 on the “normalization” of Hong Kong ruled that “the Special Administrative Region of Hong Kong (Hong Kong) is no longer sufficiently autonomous to justify differential treatment in relation to the People’s Republic of China (PRC or China) under the particular United States laws and provisions thereof set out in this order” [38].^3^ This was accompanied by measures to formally protect the liberal way of life in Hong Kong. The Hong Kong Autonomy Act (passed into law on July 14, 2020) stated that sanctions would be imposed on “foreign individuals and entities that materially contribute to China’s failure to preserve Hong Kong’s autonomy” [45].

In a nutshell, Washington’s positioning toward Beijing is obviously driven by a strategic security-economy nexus [102]. With the signing of the “Phase One Deal” on January 15, 2020 [37], the trade war between Washington and Beijing seemed to have entered a ceasefire stage. However negative views of China as an unreliable rising power, fueled again with the spread of SARS-CoV-2 as well as by the perception that Chinese information and communications technology (ICT) companies pose a threat to national (security) interests, resulted in preemptive strategies. Already in May 2019, Trump had signed an executive order banning Chinese companies such as Huawei and ZTE from the US 5G market [36]. Thus, the ceasefire did not last long. The anti-China rhetoric displayed in the US National Security Strategy and in the “Elements of the China Challenge” compiled by the Office of the US Secretary of State in November 2020 (and revised in December 2020) [63], as well as the speeches by Pompeo, document that the PRC is not framed simply as an economic competitor but rather as an antagonist player seeking to challenge American power.

The outcome of the US presidential elections in 2020 and the inauguration of the Biden administration in January 2021 did not cause any immediate modification to the US’s latest China strategy. While Biden’s inaugural speech in January 2021 stressed the need to rebuild US democracy and to secure unity (and social coherence) at the domestic level [88] (regarded as fragile, especially following the storming of the Capitol by Trump supporters), his speech at the Munich Security Conference (MSC) and his announcement that “America is back” signaled a return to the US taking a more active role in global affairs. Biden also declared that the US would rejoin the Paris Agreement and actively cooperate with international and multilateral institutions [91], thus ensuring there would not be any vacuum left in global governance that the PRC might actively seek to fill.

The US’s approach to security constellations in Asia, as outlined in its 2017 “Free and Open Indo-Pacific Strategy,” did not see significant revision. In his first statement on US foreign policy, delivered on February 4, 2021, Biden stressed that “American leadership must meet this new moment of advancing authoritarianism, including the growing ambitions of China to rival the United States and the determination of Russia to damage and disrupt our democracy” [89]—thus linking the aforementioned democracy-autocracy antagonism to a redefined, renewed global mission for the US. To reconfirm the latter’s dedication to the freedom of navigation and the protection of democracies, a few days after Biden took office, on February 4, 2021, the USS *John S. McCain* traversed the Taiwan Strait [58]. In April 2021, an “unofficial” US delegation visited Taiwan [73]; the Biden administration also announced new bargaining rounds on a trade and investment framework agreement with Taipei [64].

Under its new president, the US (re-)turned to selective multilateralism and undertook efforts to strengthen its security cooperation with its democratic allies in Asia and Europe. In March 2021, the Quadrilateral Security Dialogue (QUAD)—initiated in 2002 by Australia, India, Japan, and the US—issued a joint statement, “The Spirit of the QUAD.” This outlined the network’s shared vision of a “free and open Indo-Pacific” and of securing a “rule-based maritime order” in the East and South China Seas^4^ [92].

The positions stressed in Biden’s phone call with Xi on February 10, 2021 [90], did not indicate any major deviation from the US’s previous stance (meaning that the changes instituted under the Trump administration have not been revoked).^5^ The “New Approach to the US-China Trade Partnership” [65] presented by Katherine Tai, US Trade Representative, in October 2021 confirmed that the US would continue the general path of protecting and prioritizing its own economic interests, as initiated under Trump. Slightly earlier, in his second phone conversation with Xi on September 9, 2021, Biden had stressed, however, the need to “manage” bilateral competition and to avoid being drawn into open conflict [93].

## Mapping EUrope’s Position

How has the EU positioned itself with regard to the intensifying tensions and disputes between Washington and Beijing? Has Brussels simply copied the US’s position updates regarding Taiwan and Hong Kong, and joined in Washington’s 5G crusade against the PRC?

The release of its 2019 China strategy, entitled “EU–China: A Strategic Outlook,” marks a major shift in the EU’s positioning toward Beijing, as the paper lists a threefold perception of China as “cooperation *partner*, economic *competitor*, systemic *rival*” [23; italics added for emphasis]. The topics covered clearly illustrated the EU’s efforts to prevent internal fragmentation in the bloc’s approach to China, with the (16/17+1) summits between the PRC and a select subgroup of (16 Central and Eastern) European states (+ Greece) addressed directly. Moreover, the paper also stressed that the EU would expect China to respect human rights (with regard to, inter alia, Xinjiang) and “the degree of autonomy enshrined in the Hong Kong Basic Law” [23]. Taiwan is only mentioned in footnote 4, where the EU confirms its adherence to the One-China principle (as defined by Beijing).

Even though the United Kingdom opted to leave the EU, the legacy of Great Britain’s special relationship with its former crown colony Hong Kong is still discernible in the EU’s official diplomatic statements. As the situation in Hong Kong continued to escalate, on July 1, 2020, the EU once again expressed its “grave concerns” regarding the passing of the PRC’s aforementioned National Security Law. The related joint declaration was even officially supported by the EU’s candidate member countries (Albania, Montenegro, North Macedonia), potential candidate countries (Bosnia and Herzegovina), as well as the European Free Trade Association countries [17]. On July 22, 2020, the EU released the “Hong Kong 2019 Annual Report,” tellingly subtitled “an exceptionally challenging year.” The report highlighted the importance of upholding the “One Country, Two Systems” formula and introducing revised election modes as generally projected in Hong Kong’s Basic Law [28].

The European Parliament (EP), as well as the political (opposition) parties within EU member states, have been far more critical in their responses to Beijing’s role in and beyond Asia. Already in June 2020 the EP had released a joint motion on resolution, pinpointing that it:

“[S]upports the VP/HR’s assessment that a new and more robust strategy *to deal with a more assertive China* is necessary, as well as an open and honest dialogue; urges the Council and the EEAS *to adopt a stronger position supporting Hong Kong’s continued legal autonomy*; stresses that this is paramount to let pro-democracy supporters in Hong Kong and the wider international community know that the EU will stand by its founding values of freedom, democracy, respect for human rights and the rule of law” [30; italics added for emphasis].

The EP also underlined the threat that the National Security Law would pose to Taiwan [30]. Also, on January 20, 2021, the EP released a resolution on the implementation of the EU’s Common Foreign and Security Policy, expressing its concerns about the situation in Hong Kong, praising Taiwan’s solidarity with the EU in facing the COVID-19 pandemic, and calling on the PRC “to reach a peaceful resolution of all land and sea border disputes”—referring to Taiwan, the South China Sea, as well as to the border dispute(s) with India. With regard to Taiwan, the resolution used the framing “China’s increasingly provocative military manoeuvres aimed at Taiwan” and warned Beijing “to refrain from taking unilateral action to change the status quo” [31]. The EP also encouraged the EU and its individual member states to “revisit their engagement policy with Taiwan and to cooperate with international partners in helping sustain democracy in Taiwan free from foreign threats” and “to advocate for Taiwan’s membership as an observer of the World Health Organization and World Health Assembly, and other international organizations, mechanisms and activities, as well as of the global disease prevention network” [31]. Similar statements and recommendations can be found in the EP’s 2021 resolution on the implementation of the Common Security and Defense Policy [32].

Finally, on July 24, 2020, the Council of the EU passed a joint position statement as a response to the National People’s Congress’s passing of the New Security Law. The EU expressed its commitment to the “One Country, Two Systems” formula and voiced its “great concern” regarding the new law. It also underlined that it would continue its close cooperation with civil society in Hong Kong, observe the “trials of pro-democracy activists,” and limit “exports of specific sensitive equipment and technologies for end-use in Hong Kong, in particular where there are grounds to suspect undesirable use relating to internal repression, the interception of internal communications or cybersurveillance” [18].

Power struggles and tensions between the EU Commission and factions within the EP have also become observable, especially in connection with the Comprehensive Agreement on Investment (CAI), signed by the EU and China in December 2020 [25]. In light of the sanctions imposed on EU entities and officials—Beijing’s response to EU sanctions imposed in May 2021 on Chinese counterparts deemed responsible for human rights violations in Xinjiang—the EP refused to ratify the agreement [33].

The imagination of Taiwan as an alternative (and democratic) “Chinese” cooperation partner has also emerged as a controversial issue within the party politics of Eastern Europe. In 2020/2021, several states formally belonging to the 16+1 network—connecting the PRC with 16 countries located in Central and Eastern Europe (11 of them member states of the EU)—expressed their dissatisfaction with the implementation of the once-announced Belt and Road Initiative (BRI) cooperation projects. The number of BRI-related investment and infrastructure projects has remained far below these countries’ expectations, which has weakened the original euphoric welcoming of the PRC as an alternative to Brussels’ conditional loans and credit lines, as the case of the Czech Republic evidences [71]: in 2018, Ye Jiamin—the founder of CEFC China Energy, a privately-owned Global Fortune 500 company that had acquired ownership or shares of several well-known Czech brands—was arrested for corruption in Beijing [48]. In 2017, Milos Zeman, the Czech president, had appointed Ye as his economic adviser—the latter’s arrest thus painted Zeman’s partnering with Chinese companies in a rather negative light. When the state-owned Chinese company CITIC moved in and replaced CEFC, also paying off the inherited debts, this seemed to confirm the threat perception of a silent takeover of EUrope by Beijing via investment and debt-trap diplomacy [39].

The visit to Taiwan by Czech Senate leader Milos Vystrcil and his statement “I am Taiwanese”—reminding onlookers of the famous Berlin speech by John F. Kennedy delivered during the high tides of the Cold War—are a testament to the dividing lines running between the political parties in the Czech Republic. Even though Zeman condemned Vystrcil’s visit as “boyish provocation” approved of neither by the government nor by the president himself [69], Beijing reacted by means of retaliation and annulled an already-signed deal with the Czech Republic’s leading piano company. Zeman’s attempts to strengthen ties with Moscow and Beijing, were, as not only this Taiwan episode illustrates, contrary to the wishes of the country’s political opposition.

Moreover, apart from debt-trap scenarios and disappointed hopes of profiting from China’s BRI, Eastern and Central European societies’ views of China are also overshadowed by people’s experience and remembrance of Communist Party governance and suppression of civilian contestation movements by the Soviet Union. The increased perception of the PRC as a “socialist” state under recentralized CCP rule exporting illiberal “Chinese” governance principles has hence fueled threat perceptions and increased the pressure on the Czech government to cancel already-agreed BRI activities. The Czech intelligence agency (BIS) even warned against allowing investment (and purchases) by China and Russia. In September and October 2020, Sinophone Borderlands conducted a survey in 13 European countries to measure European views on and perceptions of China—reporting 56 percent of respondents in the Czech Republic holding unfavorable views, which implies a dramatic decline and severe deterioration of China’s image there as compared to 2017. Only North Korea and Russia received more negative ratings. Political parties’ perceptions of the PRC largely reflect their political orientation: while the communist/socialist camp largely sides with the China strategy promoted by Zeman, the pro-democratic, liberal opposition upholds the legacy of Vaclav Havel in siding with the Chinese dissident and pro-Tibet movements [8, 82].

Likewise, also in 2021, the Baltic state Lithuania formally announced its decision to leave the 16+1 network. In July 2021, Lithuania and Taiwan declared the reciprocal opening of permanent trade offices. When it was announced that the delegation office in Vilnius would be named “Taiwanese Representative Office in Lithuania”—thus indirectly signaling statehood, as in countries subscribing to Beijing’s One-China Principle the correct labeling would be “Taipei”—the PRC recalled its ambassador, asked Vilnius to recall its own from Beijing, and announced trade sanctions. The EU responded by expressing unified solidarity with its Baltic member state. The conflict escalated even further after the Lithuanian Defense Ministry began advising its citizens to refrain from buying or using Chinese smartphones, as these were said to violate data privacy as well as contain built-in software identifying and censoring words and phrases deemed “sensitive” by the Chinese government [53].

Concerning the battle over national and global 5G networks, in March 2019 the EU Commission released a recommendation on the cybersecurity of these networks. Then, on July 24, 2020, it published a report on the implementation of the EU’s 5G toolbox. Mitigating measures include the strengthening of national regulatory institutions, the evaluation of risks to national security associated with the inclusion of non-European technology suppliers, and the continued screening of foreign direct investment [29]. Contrary to the US, the EU has chosen to jointly develop technological (and ethical) standards for 5G (in close cooperation with the US and China). The wording of the EU’s document is quite neutral, refraining from any negative framing of China’s ICT plans and activities. Nonetheless, the EU has continued to work on European frameworks for standardization and regulation in the fields of 5G and artificial intelligence (AI). The “2021 EC Coordinated Plan on Artificial Intelligence,” released in April 2021, is dedicated to the ambitious goal to “create the EU’s leadership in human-centric AI” [26]—thus signaling symbolic competition with both the PRC as well as with the US.

## Perception Shifts and Polygonal Relations

The modifications of both Washington’s and Brussels’s approaches to the PRC have occurred incrementally, and are not linked to specific compositions of political elites: the toughening of the US’s China strategy can be traced back to its “Pivot to Asia” initiated under the Democrats (and specifically President Barack Obama); Republican Trump’s new Taiwan-related acts were not revoked by the incoming Biden administration. In the case of the EU, the year 2019 saw the election of a new president of the EU Commission, officially in office since December of that year. However the labeling of the PRC as a “systemic rival” in the EU Commission’s official communication happened in March 2019 already—in other words, prior to the inauguration of Ursula von der Leyen.

Both the US and EUrope have obviously responded to an increasingly negative perception of PRC politics and Beijing’s anticipated global ambitions. Over the past two decades, the PRC’s international image and reputation have continuously deteriorated, gaining momentum in conjunction with Beijing’s more active positioning on the global stage under Xi.^6^ Increasingly unfavorable views have further accelerated with the global spread of SARS-CoV-2. Pew 2020/2021 survey data reveals the following: in Australia, 81 percent of people had a negative view on China (2019: 57 percent); in Canada and the US, that figure was 73 percent [81]. A similar trend is observable in EUrope. However, individual EU member states’ popular perceptions of the PRC are quite fragmented and diverse. In Italy, a country seeking to boost its economy via joining Beijing’s BRI initiative (a Memorandum of Understanding was signed in March 2019), unfavorable views have remained at a relatively stable level of around 60 percent; positive views even increased there from 27 percent in the first decade of the twenty-first century to 32 percent by 2020.^7^ In the same period, however, in Germany and the Netherlands unfavorable views of China rose from 34 percent and 37 percent in 2005 respectively—in the UK from 16 percent—to over 70 percent in all three of these countries by 2020 [80]. These survey results evidence that Beijing’s efforts to promote a positive image of China as a peaceful and cooperative great power (*daguo*)—as stressed in official speeches and as visualized in CCTV documentaries on China’s role in world affairs (e.g., *Daguo Waijiao*,^8^ broadcast in 2017)—did finally not help to diffuse the threat perceptions associated with the Chinese Communist government.

While the reputational losses of the PRC, occurring almost simultaneously in the US and EUrope, might, at first glance, appear to be the outcome of shared values and close policy coordination, the image of the US among European countries has in fact likewise declined (Tables 1, 2, 3).

**Table 1:** Favorable Views on the US (in %)

Country	2000	2020
France	62	31
Germany	78	26
UK	83	41

Source: Pew Survey Data Summer 2020.

**Table 2:** Comparative Perceptions of Russian, Chinese, and US Presidents (in %)

President	Confidence	No Confidence
Vladimir Putin (Russia)	23	73
Xi Jinping (PRC)	19	78
Donald Trump (US)	16	83

Source: Pew Survey Data Summer 2020.

**Table 3:** Level of Trust in the US as a Reliable Partner (in %)

Country	Favorable Perceptions
Germany	51
Sweden	56
France	60
Netherlands	61
Spain	64
UK	67
Italy	73
Poland	76

Source: Transatlantic Trends 2021.

The uncertainties created by Trump’s announcement of his administration canceling multilateral agreements in the fields of global security, of global environmental as well as climate protection, and that the US mission in Afghanistan would end led to declining trust in the North American country among allies and partners worldwide. According to Pew survey data, the US president (Trump) received even lower trust scores than his Chinese and Russian counterparts.

The US’s announced withdrawal from multilateral treaties and engagement in the fields of global security under Trump had a catalyzing function, refueling the EU’s earlier quest to establish an entirely European security architecture. While the UK had always insisted on close security and defense cooperation with the US, France repeatedly stressed the need for “strategic autonomy,” leading to a significant shift in the EU’s formal defense agenda. In 2017, the European Defense Fund was established. Moreover, 25 EU member states agreed to coordinate their national armed forces (Denmark and Malta opted out) and established the so-called Permanent Structured Cooperation (PESCO). In 2018, upon the initiative of France, a European Intervention Initiative (EI2) was launched [59]. Further, French president Emmanuel Macron, a fierce promoter of European sovereignty, has called for the formation of an independent European army, pronouncing the “brain death” of the North Atlantic Treaty Organization (NATO) [86]. Even after Biden’s official commitment to multilateral actions in 2021, Macron insisted on building a more independent EU security and defense architecture, underlining that only a more autonomous EU—in terms specifically of military and self-defense capacities—would be able to act as a reliable partner to the US [74]. The EU’s efforts to upgrade its foreign security and defense strategy—lifted to a higher level of integration by the Treaty of Lisbon (2009) and further strengthened by the passing of the European Union Global Strategy (2016)—did not, however, respond only to the position adjustments of the US or to a perceived increase in China’s global presence. As reassessments of the background to the European Security Strategy (2003) and the drafting of the EU’s Global Strategy (2016) evidence, the EU’s strategy papers were developed to overcome internal rifts and frictions among and within member states against the wider backdrop of both regional and global turbulence (Color Revolutions, Eurozone crisis, Arab Spring, Brexit) [3, 83, 96].

It is obvious that EUrope is not planning to blindly copy US positions vis-à-vis China. The chaos caused by the withdrawal of US troops from Afghanistan in August 2021 (implying also the end of Europe’s military engagement in that country) is perceived as a partly irresponsible act, leaving EUrope with new security dilemmas. Moreover, the release of the joint statement “The Spirit of the QUAD” not only sent a clear signal to the PRC but also illustrated that the US would seek to multiply its security alliances and not exclusively rely on cooperation with EUropean partners. In September 2021, Australia, as noted a QUAD member, unilaterally canceled an already-existing deal to purchase French submarines (signed in 2016) and instead jointly with the US and the UK formed the trilateral AUKUS security pact, which also included a new deal providing Australia with nuclear-powered submarines. France responded by recalling its ambassador [105], perceiving this act not only as diplomatic affront but also as detrimental to its own efforts to promote an alternative strategy for the Indo-Pacific beyond the initiatives coordinated by Washington or Beijing. Back in 2019, France had released its own Indo-Pacific strategy (updated in 2021) [60]; Germany published its own principles and guidelines in 2020. Both countries are reportedly pushing the EU to adopt a unified stance on the Indo-Pacific [61].

Transatlantic Trends 2021, a joint project by the German Marshall Fund and the Bertelsmann Foundation, reveals that the level of trust and confidence in the US as a reliable partner varies across the EUropean countries: from 51 percent in Germany, to 60 percent in France, to 76 percent in Poland [97].

The level of trust in and reliance on the US is obviously comparatively high in some of the Baltic states and certain parts of Eastern Europe, regions which also show a strong dedication to NATO. This, however, might result less from any immediate threat posed by China but reflect rather Russia’s intervention in Ukraine (and the annexation of Crimea), leading to threat perceptions among those former member states of the USSR with a large proportion of ethnically Russian people. This also explains these countries’ willing support of US interests in symbolic exchange for security guarantees. In December 2019, in the run-up to the earlier-mentioned CAI, Polish foreign minister Zbigniew Rau underlined the need to consult with the US and to strengthen transatlantic policy coordination [72]. This, shortly afterward, was also demanded in a Twitter statement by then-incoming US National Security Adviser Jake Sullivan [19]. A European Council on Foreign Relations survey conducted after the US elections documents that Poland sees the US as its most important partner (45 percent), far ahead of its neighbor state Germany (29 percent). 77 percent of people in Poland are convinced that their security interests will only be safeguarded and protected by US security guarantees (i.e. far above the European average of 57 percent) [9]. There is obviously a causal connection between a European country’s views on the US—as potential provider of security and protection—and its positioning vis-à-vis the latter’s China strategy. Given that the US security and defense strategies name the PRC, Russia, as well as other autocratic regimes (such as North Korea) in the same breath, siding with the US to secure protection against Russia implies symbolic compliance with Washington’s rediscovered alliance with Asian democracies.

## Democratic Global Power Identities and Ontological Security

One of the noticeable parallels between the US and EU narratives on the PRC is the reactivation of the rival image as a new subtype of the old enemy image. The framing of China (and Russia)^9^ as the promoter(s) of an illiberal global order and unreliable player(s) in US and European foreign politics, fueled by the negative reputation of the PRC in connection with the global spread of the COVID-19 pandemic [50, 56], echoes the enemy-image-driven security spirals of the Cold War era. As analyses of the latter’s conflict constellations document, enemy images function as self-fulfilling prophecies: information that would contradict these stereotyped classifications of states as good or evil (enemy) ones is generally ignored [44], which leads to a never-ending reconfirmation of (mis)perceptions and aggravates security dynamics [51]. In this vein, Wang Ziyuan’s study identifies a (global) status dilemma, fueled by misperceptions, as the main factor triggering the deterioration of US–China relations [101].

The framing of others as “the enemy” is closely connected to self-images and role identities. Studies departing from the basic assumptions of social-identity theory—as developed by Giddens [40, 41] and Robert David Laing [55]—postulate that actors generally engage in (symbolic) actions that reconfirm their established role identity, such as the US’s formation of alliances with democracies in Asia or the EU’s support for democratic resilience in its immediate neighborhood. 10

US and EU reactions to domestic rifts and external economic and security challenges—reflecting regional and global crises not necessarily directly caused by the PRC—have resulted in a reevaluation and modification of both players’ self-images as defenders of democracy and the liberal order. Via the framing of external others—namely the PRC (and Russia)—as acting in opposition to these principles, both players seek to restore their ontological security—weakened by the rise of populist, antidemocratic movements at home. The rediscovery of “democratic” role identities hence enforced the symbolic “barbarizing” of China [110] (and Russia) as “autocratic” global competitors.^11^

While the readjustments (and partial reconfirmations) of US and EU role identities would occur almost simultaneously but not as directly synchronized forms of strategy coordination, the parallel rediscovery of democratic role-identity patterns has catalyzed the renewal of strategic transatlantic cooperation. In December 2020, the month when, as noted, the EU and China signed the CAI, the US and the EU agreed on “A New Transatlantic Agenda for Global Change” that established a new EU–US Dialogue on China [24]. However, this does not imply that the EU would unanimously follow Washington’s approach to Beijing, as the formulation of Indo-Pacific strategies by France, Germany, and the Netherlands—and their lobbying for a joint EU position [61]—illustrate. Likewise, under Biden, the US has multiplied its foreign relations and currently operates on the basis of selective multilateralism. Moreover, as his speech at the 76th session of the UN General Assembly makes clear, the US has started to coin its own narrative of world order, presented not as a dream for a distant future but as a restoration of the old order—“Building Back Better World” [94]—that counters the Chinese global charm offense of the “China Dream” and the “New Silk Road.”^12^

Following Biden’s reconfirmation of transatlantic (security) cooperation at the MSC in February 2021 [91], transatlantic coordination in the fields of security and defense was then addressed at the NATO summit in Brussels in June of the same year. The joint declaration of the participating heads of states and governments noted how, as an alliance, they would jointly respond to the challenges China’s global rise “can present” and continue a joint strategy of “engaging” the East Asian country. Both the PRC and Russia were labeled “assertive” and referred to as challengers to the rules-based international order [62]. Despite NATO chief Jens Stoltenberg’s statement that “we’re not entering a new Cold War and China is not our adversary, not our enemy” [4], the enemy/rival image has obviously substituted earlier wishful ideas of successfully transforming China into a democracy. Shared perceptions and value orientations have been rediscovered as the binding glue of transatlantic cooperation, symbolically expressed by Stoltenberg when meeting with Biden prior to the NATO summit in Brussels—“China’s leaders don’t ‘share our values’” [35]—thus dividing the world, once again, into two antagonistic camps.

The US and the EU share many interests in the fields of (free) trade and have confirmed their joint interest in coordinating their response to unfair Chinese trade practices—as documented in their “US-EU Trade and Technology Council Inaugural Joint Statement” (September 2021) . Interestingly, this transatlantic accord reflects on trade issues in connection with technology. The latter is linked also to the revived democracy-autocracy antagonism by their stressing of a joint dedication to “AI systems that are innovative and trustworthy and that respect universal human rights and shared democratic values” [27].

## Conclusion

The past few years have witnessed a revival of open power competition, often compared to the system antagonism of the Cold War [103]. Contrary to expectations, a new strategic triangle— this time composed of China, EUrope, and the US [78]—did ultimately not materialize. As this article has shown, given the increasing internal fragmentation of the EU into various subgroups of states with competing views and positions vis-à-vis both Washington *and* Beijing, instead of a strategic triangle this complex tripartite relationship would be best descriped as a polygonic one—mirroring the variety of EUropean actors and interests involved. Furthermore, while Washington’s strategy is based on the perception of China as “strategic competitor,” the EU’s threefold role ascriptions of “partner, rival, competitor” allow for cooperation with China in select policy fields while pursuing independent and autonomous positioning in those where national security interests are concerned. In this vein, Yves Tiberghien has summarized the EU’s China strategy as a combination of “multilateral reinforcement, Cold War avoidance, and complex balancing” [95].

Overall, the findings presented here reconfirm the results of the earlier study by Riddervold and Rosén [75]. Analyzing similarities and divergences in the US’s and the EU’s positioning in light of the crises and conflicts in the Ukraine and the South China Sea, they conclude by stressing that the EU is increasingly developing its own approach to foreign and security affairs and would be less willing to rely on a sometimes unpredictable US partner (their study mainly deals with the Trump years). They hence summarize their main finding as the emergence of “a stronger and more autonomous EU in a weaker EU-US relationship” [75:567]. Their study, however, could not foresee the internal fragmentation and centrifugal tendencies within the EU and the inclination of some member states to rely on the US as external protector as well as to maintain strategic distance from European security and defense initiatives promoted by France—dynamics gaining momentum in the aftermath of the US presidential elections of November 2020.

In a nutshell, the rediscovery of the democracy-autocracy divide in world affairs and the spread of a negative “re-autocratizing” China image (in connection with the narratives of debt trap-diplomacy and of China being the main culprit for the global COVID-19 pandemic) have catalyzed the revival of the transatlantic network of democratic allies—establishing a new consensus among democratic parties regardless of their political hue. In the direct aftermath of the parliamentary elections in Germany, surveys documented a silent consensus across *all* political parties that Berlin should pursue a tougher stance on China [49]. Moreover, survey results from 2020/2021 presented by the Körber Foundation show that 82 percent of people in Germany think their country should take a neutral position between Washington and Beijing, while 51 percent would like to see Germany and Europe become less dependent on the US and contrariwise 41 percent would opt for continuing transatlantic cooperation. Further, 78 percent believe that transatlantic relations will normalize under the presidency of Biden [54].

The emerging distrust of the (former) 16+1 network member states in BRI cooperation and the rising security concerns in the fields of AI and ICT all across the continent have triggered new EUropean and transatlantic “democratic” harmonization in facing the rise of Chinese “autocracy” (and assumed “autocracy promotion”). EUrope is far less united, however, with regard to its position vis-à-vis the US and the latter’s visions of regional and global security arrangements.

As 2020 surveys on public perceptions of China and the US in EUrope evidence, there is a widely shared belief that the relationship with the PRC is mainly based on economic interests, whereas transatlantic relations are considered as being in EUrope’s political as well as economic interests and to rely on shared norms and values [5]. However, while the EP’s debate on a “new” EU China strategy [34] and the speech delivered by the EU’s high representative Josep Borrell at the EP [22] on September 14, 2021 underlined that the EU would not formally deviate from the threefold role-ascriptions towards the PRC as “partner, rival, competitor,” the internal debate speaks of China as an “increasingly assertive global power” representing values incomplatible with EUropean norms and democratic principles [22]. The EP’s debate, however, also referred to Afghanistan to underscore the need for a EUropean army – hence signalling the EP’s general dedication to the principle of strategic autonomy within the global strategic polygones [34].

**Fig. 1.**
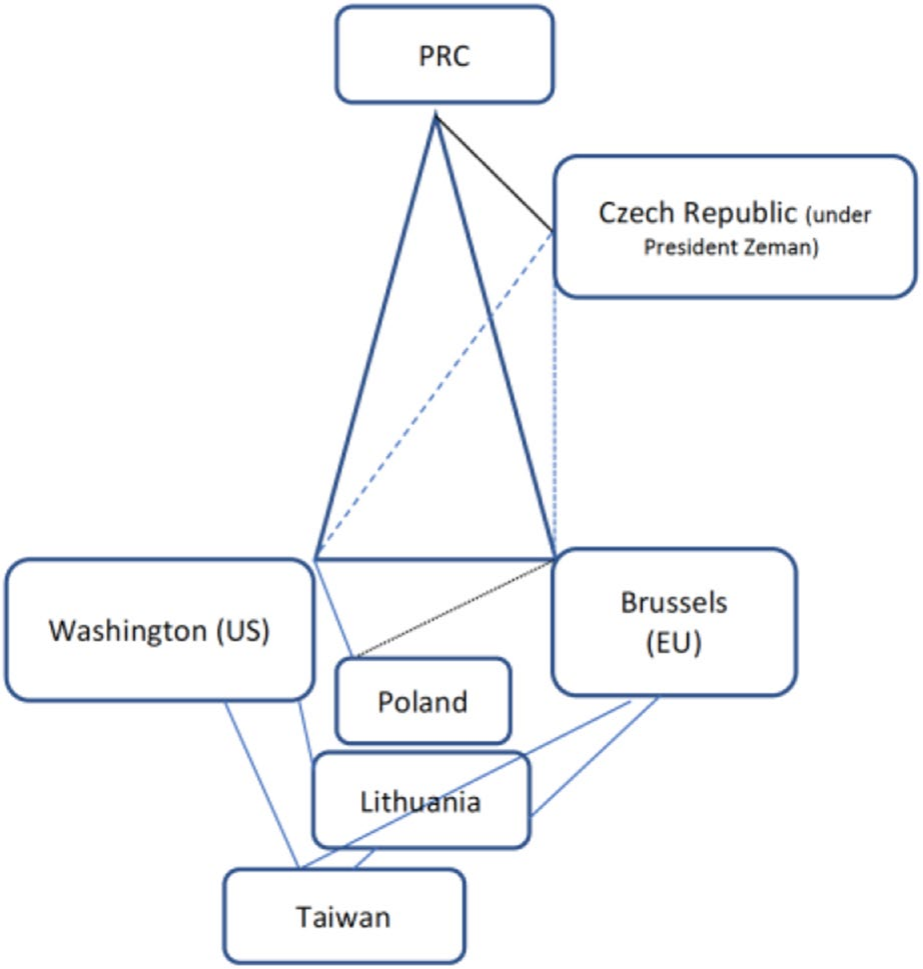
Strategic Polygones

As this article has shown, shared norms and values have a positive effect on players’ willingness to engage in cooperation or in the formation of joint alliances. Taking the factors of power asymmetry and normative proximity/distance into account, this article suggests a further modification of the strategic triangle that also reflects the recently re-emerging division of the world into democratic and autocratic political regimes—a grouping symbolically underscored by the US’s Summit for Democracy (December 2021), with Taiwan listed as an independent participant—and the thereform resulting implications for strategic poly-angulation. As fig. 1—summarizing the examples referred to in this article—illustrates, the EU as (imagined) unitary actor plays a pivotal role in an isosceles triangle, with both Brussels and Washington expressing a certain normative distance towards the PRC. The EU’s member states, however, sometimes deviate from Brussels’ official positions, causing the fragmentation of the triangle into dynamically fluctuating polygones.

## Footnotes

1 This term refers to both the European Union as supranational actor as well as to the EU’s individual member states. The EU’s internal fragmentation has been aggravated by the spreading coronavirus: EU member states (re-)started to prioritize the principle of national sovereignty and border security [57, 76]

2 Relational power, including symbolic positions, perceptions, as well as mutual role ascriptions is a powerful driver of China’s foreign diplomacy, more powerful, as some authors argue, than mere material power capacities [1].

3 These laws and executive orders ended the special status granted to Hong Kong by the US via the Hong Kong Policy Act (1992). In 2019 already, the US had passed the Hong Kong Human Rights and Democracy Act (2019).

4 On the symbolic meaning of the South China Sea and implications for legitimacy: [46].

5 In January 2021 the United States Institute of Peace hosted a discussion with the national security advisers to the Trump and Biden administrations, documenting the silent consensus between the US’s political camps regarding Washington’s China (and Asia) strategy [100].

6 Beijing’s efforts to upgrade its discursive power did hence not lead to the expected results [10, 12]. By contrast, the active promotion of “Chinese” frames and narratemes at the global level even added to the perception of an increasingly “assertive” behaviour [77].

7 As Chen’s evaluation of the media coverage of Beijing’s mask and medical cooperation diplomacy documents, Italy was the only EU country where this initiative resulted in a positive perception of China [13].

8 For a theory-guided analysis of *Daguo Waijiao*, see: https://www.worldorderfluctuations.net/dfg-research-project/.

9 China’s strategic moves in Central Asia illustrate that Beijing is seeking to avoid any open confrontation with Russia while expanding its own economic footprint in Moscow’s former sphere of control [70]. So far, however, there is no empirical evidence for the existence of a strategic axis of policy coordination (and joint autocracy promotion) between Beijing and Moscow.

10 Recent studies have identified uncertainties and ontological insecurity in light of the COVID-19 pandemic as the main accelerator of Australia’s tough-on-China policy (and its strengthening of security and defense cooperation with the US and Asian democracies) [66].

11 At the global level, the revival of the democracy-autocracy divide might, ultimately, result in a fragmentation of the international order into competing normative networks clustered around rising regional power centres [52].

12 For a role theoretical reading of Sino-US relations: [79].
